# Firm, yet flexible: a fidelity debate paper with two case examples

**DOI:** 10.1186/s13012-024-01406-3

**Published:** 2024-12-05

**Authors:** Bianca Albers, Lotte Verweij, Kathrin Blum, Saskia Oesch, Marie-Therese Schultes, Lauren Clack, Rahel Naef

**Affiliations:** 1 Institute for Implementation Science in Health Care, Universitätstrasse 84, Zurich, 8006 Switzerland; 2 Center of Clinical Nursing Science, University Hospital Zurich, Zurich, Switzerland; 3 Division of Infectious Diseases and Hospital Epidemiology, University of Zurich and University Hospital Zurich, Zurich, Switzerland

**Keywords:** Implementation science, Fidelity, Adaptation, Intervention adherence, Implementation strategy

## Abstract

**Background:**

In healthcare research and practice, *intervention* and *implementation* fidelity represent the steadfast adherence to core components of research-supported interventions and the strategies employed for their implementation. Evaluating fidelity involves determining whether these core components were delivered as intended. Without fidelity data, the results of complex interventions cannot be meaningfully interpreted. Increasingly, the necessity for *firmness* and strict adherence by implementers and their organizations has been questioned, with calls for flexibility to accommodate contextual conditions. This shift makes contemporary fidelity a balancing act, requiring researchers to navigate various tensions. This debate paper explores these tensions, drawing on experiences from developing fidelity assessments in two ongoing effectiveness-implementation hybrid trials.

**Main body:**

**First**, given often scarce knowledge about the core components of complex interventions and implementation strategies, decisions about fidelity requirements involve a degree of subjective reasoning. Researchers should make these decisions transparent using theory or logic models. **Second**, because fidelity is context-dependent and applies to both interventions and implementation strategies, researchers must rethink fidelity concepts with every study while balancing firmness and flexibility. This is particularly crucial for hybrid studies, with their differing emphasis on intervention and implementation fidelity. **Third**, fidelity concepts typically focus on individual behaviors. However, since organizational and system factors also influence fidelity, there is a growing need to define fidelity criteria at these levels. **Finally**, as contemporary fidelity concepts prioritize flexible over firm adherence, building, evaluating, and maintaining fidelity in healthcare research has become more complex. This complexity calls for intensified efforts to expand the knowledge base for pragmatic and adaptive fidelity measurement in trial and routine healthcare settings.

**Conclusion:**

Contemporary conceptualizations of fidelity place greater demands on how fidelity is examined, necessitating the expansion of fidelity frameworks to include organizational and system levels, the service- and study-specific conceptualizations of intervention and implementation fidelity, and the development of pragmatic approaches for assessing fidelity in research and practice. Continuing to build knowledge on how to balance requirements for firmness and flexibility remains a crucial task within the field of implementation science.

Contribution to the literature

- Our contemporary understanding of fidelity in delivering and implementing complex healthcare interventions has broadened. Fidelity involves a balancing act between firm adherence to key principles and the need for flexibility.
- We explore the implications of this tension between firmness and flexibility, using two ongoing hybrid effectiveness-implementation trials as illustrative examples.
- We provide recommendations for navigating this tension inherent in fidelity concepts and suggest future directions for strengthening contemporary fidelity research.

## Background

### Fidelity revisited

Fidelity continues to be a widely discussed concept in complex intervention research, implementation science, and other disciplines focusing on delivering research-supported health interventions in practice. Fidelity has traditionally been defined as “*the degree to which (…) program providers implement programs as intended by the program developers*” [1] (p.240) and is the most frequently assessed process outcome in intervention and implementation research [2]. Its assessment seeks to identify elements of interventions that have been delivered and used as intended, e.g., as outlined in an intervention or program manual. Outcomes of complex interventions cannot be meaningfully interpreted without fidelity data, especially in multicomponent intervention research or in the absence of evident effectiveness [3, 4]. In this sense, “*fidelity acts as a potential moderator of the relationship between interventions and their intended outcomes*” [5] (p.1), i.e., it affects the strength or direction of this relationship [6, 7]. Moreover, clear fidelity conceptualizations allow for the specification and study of mechanisms through which implementation and intervention processes are anticipated to impact clinical outcomes and achieve desired results, thereby helping to test and validate the effective core components of health interventions [8].

Multiple frameworks exist that capture essential elements of intervention fidelity. Carroll et al. (2007) [5] developed an influential conceptual framework that centers on *adherence* as “*the bottom-line measurement of implementation fidelity*” (p.4). Its constructs reflect common elements that also characterize other fidelity frameworks [1, 3] including: (1) *adherence* or *integrity* in delivering an intervention as described in a protocol; (2) *dose* or *exposure*, describing the quantity or frequency of the intervention delivered by providers or received by end-users; (3) providers’ *quality* of intervention delivery; (4) participant *responsiveness*, characterizing participants’ engagement in the intervention; and (5) program *differentiation*, i.e. the degree to which intervention elements to be implemented are substantially different from those characterizing other interventions already in use in the local context. With these commonalities in mind, this first generation of fidelity frameworks has a strong focus on *intervention* fidelity.

More recently, this focus has shifted toward greater attention to *implementation* fidelity. By implementation, we mean the intentional and systematic selection, design, and use of strategies for enhancing the quality with which a complex intervention is integrated into and used in routine healthcare settings [9]. Accordingly, *implementation fidelity* describes the degree to which implementation strategies for integrating research-supported interventions into routine service settings are applied as intended or outlined in an implementation plan or manual [10].

This is reflected in a recently developed interdisciplinary framework, which integrates *intervention* and *implementation* fidelity into a comprehensive conceptualization of fidelity in healthcare studies [10]. Its developers assume that both intervention and implementation fidelity moderate the relationship between clinical interventions and patient outcomes – and in particular, the relationship between implementation strategies and implementation outcomes, i.e., the degree to which a complex intervention is perceived as feasible, acceptable, appropriate and can be adopted, achieve penetration and be implemented as intended [4]. Hence, a better understanding of whether implementation strategies are applied as intended or undergo adaptation will provide additional insight into why observed study outcomes were obtained or failed to be actualized.

While intervention and implementation fidelity are clearly distinguished in this framework, both fidelity types share the same ten dimensions, six (adherence, differentiation, quality, timeliness, dosage, and adaptation) relating to the fidelity of *implementers*, i.e., those delivering interventions and implementation strategies, and four (reach, enactment, responsiveness, and exposure) to that of *receivers*, i.e., patients, family members, and other users of healthcare services.

Compared to the previously introduced fidelity dimensions, this framework adds *timeliness*, i.e., the degree to which an intervention or implementation strategy is applied at the right time, *adaptations* made to an intervention or implementation strategy, and *enactment*, i.e., whether receivers comprehend and comply with implementer-suggested activities as further dimensions to consider when assessing fidelity. Furthermore, this framework introduces a novel differentiation between implementer and receiver perspectives on fidelity and emphasizes that fidelity should be assessed at two levels, that of the intervention and that of the implementation strategy. This contributes to greater conceptual clarity in a field where *implementation* fidelity continues to be used as a synonym for what, from an implementation science perspective, should be more correctly labeled *intervention* fidelity [11]. The distinction between implementation and intervention fidelity forms the basis for the remainder of this paper.

### Beyond firmness – the call for flexible fidelity concepts

Conceptually, intervention and implementation fidelity call for a degree of *firmness* in that intervention providers are expected to display a minimum of adherence or compliance in their activities to ensure intervention and implementation integrity.

In contemporary healthcare research, most *interventions* being studied are *complex*, i.e., contain several interacting behavioral, technological, or organizational components, which are delivered in dynamic clinical or community environments [12, 13]. Most complex interventions need continuous adaptation to fit local contexts, optimize delivery, and support uptake. Classical dimensions of *intervention fidelity*, however, do not address intervention adaptation while maintaining its effectiveness [14]. This gap has been acknowledged as a challenge in the literature [15], among others, leading to the development of frameworks to support adaptation tracking and decision-making in intervention [16–19] and implementation strategy use [20]. However, as highlighted elsewhere [21], these tools require expertise and resources that often do not exist in practice settings. Hence, combining these two concepts continues to be challenging because they reflect two contrasting principles [1, 14], with *fidelity* representing the intention to deliver a studied intervention precisely by protocol and *adaptation* representing the need to deviate from this protocol. This is a pertinent tension for clinical practice, where the principle of “one size does not fit all” prevails. Here, continuous intervention adaptations are required to fit local implementers’ and users’ needs [13, 14, 21, 22] and have been shown to lead to more fitting and effective interventions [22]. Still, the debate on acceptable intervention adaptation while upholding effectiveness remains unresolved [23, 24].

Attempts have been made to overcome this limitation of traditional fidelity concepts and frameworks. Perez et al. (2016), in a modified conceptual framework building on Carroll et al.’s (2007) work, have proposed to assess both intervention *fidelity* and intervention *adaptation* instead of measuring intervention adherence alone. They include two additional steps within their framework. First, to develop intervention-specific descriptors of adaptation, and second, to determine to what extent the adaptations identified affect an intervention in its entirety or some of its effective core components [14]. The modified framework has, however, not been empirically tested and validated in practice. Other scholars have developed recommendations for researchers to better incorporate adaptation processes in fidelity evaluation, e.g., by pre-defining fidelity components to be investigated or developing succinct measurement tools [25, 26]. Despite these advancements and considerations about adaptive intervention fidelity, there remains a lack of validated measures for its assessment.

While debates on *implementation* fidelity are in their infancy still, similar challenges with assessing implementation strategy fidelity and balancing firmness with flexibility have begun to emerge. Based on a scoping review, Slaughter et al. (2015) criticize the insufficient attention paid to implementation quality, reflected in a general underreporting of implementation fidelity information in healthcare studies [27]. Toomey et al. (2020) highlight that strategies used to enhance fidelity in health behavior change studies, such as manual use or standardized training and coaching, often remain unreported, thereby providing little insight into how such strategies are enacted and contribute or prevent fidelity to be built [26]. Similar conclusions were drawn by Chen et al. (2024), who, in a recent systematic review of studies of implementation strategies used in suicide prevention [28], highlighted that implementation strategy fidelity was assessed in only three of 32 included studies. Moreover, clearly differentiating between clinical intervention activities and the implementation strategies applied to make these actions happen remains challenging for researchers [28–30], further limiting opportunities to contemplate implementation fidelity-related phenomena in healthcare studies.

The implicit argument evident in this discourse is that detailing implementation strategies through transparent descriptions of the who, what, and how of strategy enactment is a precondition for understanding implementation fidelity indicators and for considering needs for implementation strategy adaptation [31, 32]. Furthermore, specifying the core components of an implementation strategy would facilitate its measurement, particularly when the “how” contains a stipulation of which content should be delivered for how long and with which intensity [21, 33].

Unsurprisingly, few studies exist that focus on measuring implementation strategy fidelity [34–37]. In a randomized trial of an organizational process improvement intervention applied in mental healthcare settings, Stein et al. (2023) developed an indicator-based, self-developed measure to assess fidelity to implementation strategies such as coaching, formal commitment from community partners, academic partnerships, or local implementation teams [35]. The development of the measure was based on a consensus process involving all research team members. Responsiveness and adherence to the strategy, as well as quality of intervention delivery, were measured using four to six indicators per fidelity dimension, with the fidelity measure being applied multiple times throughout the study. In reflecting on their fidelity measure, the authors point to an inherent risk of bias due to indicators being partly self-reported and a lack of indicators accessible and usable for a fidelity assessment, potentially limiting measure quality. This indicates common challenges and complexities in implementation fidelity measurement, including a lack of validated implementation fidelity tools and difficulties in capturing the complexity of implementation strategies in single measures [33, 38]. Furthermore, little debate has occurred about the inherent tension between a call for implementation fidelity and the commonly made assumption that implementation effectiveness depends on strategies that match the ever-changing context surrounding an implementation. This tension is particularly present in implementation trials and hybrid studies [39], where the testing of implementation strategies is a primary aim.

These examples raise two central fidelity questions. First, how can fidelity to adaptive complex health interventions be operationalized and assessed to ensure that interventions are applied as intended while creating an optimal fit between the intervention, its users, and the setting in which it is delivered? Second, how can implementation strategy fidelity be conceptualized, operationalized, and assessed while allowing implementation activities to be responsive to changing contextual conditions?

In the following, these questions will be discussed in light of two currently ongoing healthcare studies.

## Firmness with flexibility – early experience from two ongoing trials

### Intervention fidelity: the FICUS trial

FICUS (*Family support intervention in Intensive Care UnitS)* is a hybrid effectiveness-implementation study (type 1) in 16 Swiss intensive care units (ICUs)^1^. Its primary aim is to assess the clinical effectiveness of a multicomponent nurse-led family support intervention (FSI) compared to usual care using a cluster-randomized controlled trial [40] while also investigating the implementation process and outcomes using a mixed-methods multiple case study approach [41].

**Fig. 1:**
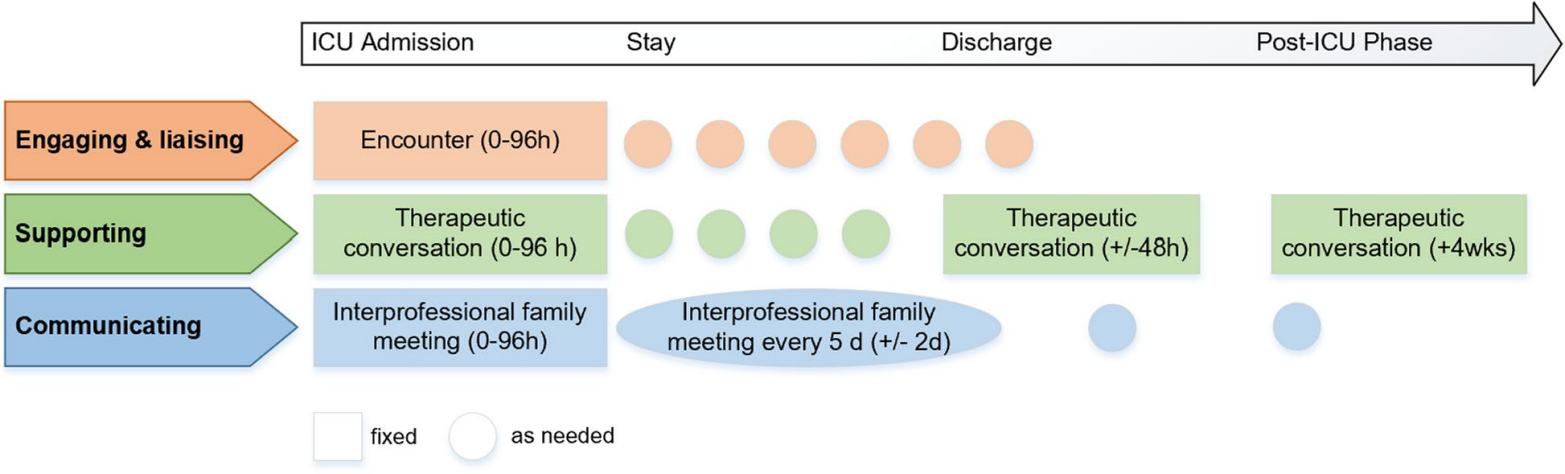
The FICUS family care pathway. This figure is unadapted from its original version and licensed under a Creative Commons Generic License (CC BY 4.0 OA). It is attributed to Naef et al. [40]

The FSI is led by trained ICU family nurses, a new role introduced to the ICU team in an interprofessional setting to support family members of critically ill patients during and after an ICU stay. It aims to improve family members’ service and (mental) health outcomes [40]. The FSI includes three core components, which are grounded in a family system nursing approach, based on evidence on systematic family interventions and existing guideline recommendations on ICU family care, and specified in a logic model (see Fig. 1 in [40]). (1) *Engaging and Liaising* (encounter) by relationally engaging families over time, connecting family with team, and coordinating and ensuring access to and involvement in care; (2) *Supporting* (therapeutic conversations) by assessing families’ situations and their preferences and needs, choosing and performing relationship-focused and psycho-educational interventions; and (3) *Communicating* (family meetings), including understanding values and priorities, informing about the patients’ conditions, discussing care plans and making joint decisions [40] Fig. 1.

**Fig. 2:**
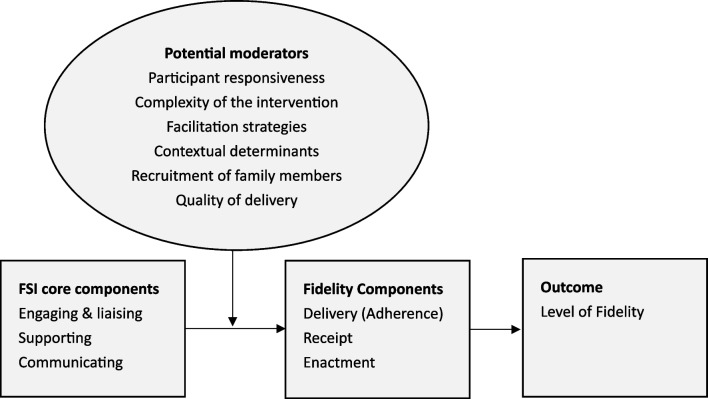
Adapted Conceptual Framework for Implementation Fidelity in the FICUS trial. This framework was adapted from the original framework by Carroll et al. 2007 [5, 30]

The core FSI components are standardized in the patient pathway but can be tailored to the patients’ courses of illness and families’ needs during and up to three months after patients’ ICU discharge. Therefore, FSI core activities are defined in a fixed and compulsory intervention dose. Family nurses adapt this dose, its frequency, and intervention components and activities to meet family members’ needs during the FSI pathway (Fig. 2).

The aim of the FICUS trial is an adaptive intervention delivery to optimize FSI fit for every family member and family as a whole and to enhance the likelihood of reaching intended service and (mental) health outcomes. To understand how intervention fidelity, the trial’s main implementation outcome [41], influences intended FSI outcomes and explains its effects, we pre-defined the minimal FSI dose hypothesized to reach causal inferences. This minimal intervention dose limited the family nurses’ room for adapting FSI to individual family members’ needs. This highlights the instant dilemma in complex intervention research between a need for adaptive interventions on the one hand and minimum intervention standards on the other, among others to evaluate causal inferences between intervention (dose) and outcomes.

#### FSI fidelity

To measure intervention fidelity to the FSI pathway and core activities, a modified version of the Conceptual Framework for Implementation Fidelity developed by Carroll et al. [5] was used. The framework was supplemented with the concepts of the three fidelity domains outlined by Bellg et al. [42], i.e., intervention delivery (adherence), receipt, and enactment (Fig. 2). *Delivery* captures whether the intervention components were delivered as intended, i.e., consistent with content, frequency, duration, and coverage criteria. *Receipt* refers to the “*degree to which the participant understands and demonstrates knowledge of and ability*” to use an intervention (i.e., comprehension), and *enactment* pertains to “*the degree to which the participant applies the skills learned*” [42] (p.444), i.e., intervention activities [25, 42, 43].

In operationalizing FSI fidelity, moderators included in the Carroll et al. (2007) fidelity framework were considered and delineated as follows [5, 41, 42]:

- *Fidelity delivery* was defined as family nurses’ adherence to core FSI activities and timeline, and the consistency of delivery across the eight intervention ICUs and between family nurses. Fidelity delivery included intervention consistency, frequency, dose, and availability (Table 1). It was assessed using an electronic structured intervention log completed by family nurses after each intervention session and by a self-reported capacity assessment measured three to six months after implementation start [41].

**Table 1:** Operationalization of fidelity delivery in the FICUS trial

Fidelity Delivery	Operationalization	Measurement
***Consistency***	Intervention delivery is defined as consistent if the minimal intervention contact dose according to the protocol was provided within the specified timeframe (i.e., five contact doses, representing all three intervention components within specified timeframe, see Fig. 1): • n (%) consistent / inconsistent delivery Intervention delivery is comparable in terms of frequency (i.e., number of interventions per patient length of ICU stay) and dose (minutes per length of ICU stay) among intervention nurses / ICUs (percentiles): • Low fidelity: 0–50 (variance > = 50%) • Moderate fidelity: 51–79 (variance between 21–49%) • High fidelity: 80–100 (variance of 0–20%).	Intervention Log
***Frequency***	Total number of interventions (conversations) per patient length of ICU stay: • Low fidelity: one intervention up to every 7th day or less • Moderate fidelity: one intervention up to every 4th to 6th day • High fidelity: one intervention up to every 3rd day. Number of interventions overall, per component, per intervention activity: • Low fidelity: dose of 2 or less interventions representing two or less components = low fidelity • Moderate fidelity: dose of 3–4 interventions representing two to three components • High fidelity: dose of 5 or more interventions representing three components.	Intervention Log
***Dose***	The total duration of interventions (conversations) divided by patient length of ICU stay among nurses / ICUs and the total duration of interventions (conversations) per component/ intervention activity (percentiles): • Low fidelity: 0–50 (variance > = 50%) • Moderate fidelity: 51–79 (variance between 21–49%) • High fidelity: 80–100 (variance of 0–20%).	Intervention Log
***Availability***	Intervention nurses’ capacity assessment for intervention delivery • Low fidelity = nurse is available 2 days or less per week • Moderate fidelity = nurse is available 3–4 days a week or when needed • High fidelity = nurse is available 5 days a week or when needed	Intervention capacity assessment, assessed 3–6 months into implementation.

- *Fidelity receipt* was defined as the degree to which family nurses understand and perform the FSI, including required cognitive and behavioral skills. It was operationalized as family nurses’ participation in educational implementation strategies targeting FSI comprehension, e.g., attendance of intervention training and monthly case conferences used to enable them to perform the intervention [40, 44]. Additionally, the performance of cognitive and behavioral intervention skills was self-assessed with the psychometrically validated German version of the Family Nursing Practice Scale [45, 46] and in focus group interviews (Table 2). As such, fidelity receipt was measured for the intervention and one specific implementation strategy.

**Table 2:** Operationalization of fidelity receipt in the FICUS trial

Fidelity Receipt	Operationalization	Measurement
Attendance	Intervention nurses’ participation rate at case conference per ICU. • Low fidelity: 0–50% attendance • Moderate fidelity: 51–79% attendance • High fidelity: 80–100% attendance.	Structured participation form.
Skills	Family nurses’ appraisal of practice skills (percentiles): • Low fidelity: 0–50 (variance > = 50%) • Moderate fidelity: 51–79 (variance between 21–49%) • High fidelity: 80–100 (variance of 0–20%).	German version of the Family Nursing Practice Scale, assessed at baseline, between 3–6, 6–9 months into implementation and after implementation completion.
Comprehension	Skills in working systemically with families.	Focus group interviews after implementation completion.

- *Fidelity enactment* was operationalized as family nurses’ engagement and performance of FSI core activities. For standardization purposes, a fidelity audit observation tool and a self-rating tool for family nurses were developed, both based on the FSI protocol [40]. The fidelity audit tool consists of four items on available structures, such as study information and 59 FSI activities alongside the three main FSI components *engaging and liaising*, *supporting* and *communicating* [41]. Observers rate family nurses’ performance during FSI activities as *performed*, *partially performed*, *not performed*, or *not applicable*. The family nurses’ self-rating tool includes three subscales, one for each FSI component (*engaging and liaising*: 15 items, *supporting*: 27 items, *communicating*: 16 items), all rated on a 5-point Likert scale. A lower score indicates lower, and a higher score higher, self-perceived FSI performance (Table 3).

**Table 3:** Operationalization of fidelity enactment in the FICUS trial

Fidelity Enactment	Operationalization	Measurement
Engagement and performance of intervention activities	The quality and consistency of intervention performance (overall and per intervention component). Level of fidelity (percentiles): • Low fidelity: 0–50 (variance > = 50%) • Moderate fidelity: 51–79 (variance between 21–49%) • High fidelity: 80–100 (variance of 0–20%).	Fidelity Audit Observation Tool, used once between 6–9 months into implementation. Fidelity Self-Rating Tool, used between 6–9 months into and after implementation completion.

In the FICUS study, using a conceptual fidelity framework enabled a nuanced and comprehensive operationalization of fidelity beyond a simple “delivery as intended”, accounting for FSI having been designed as an adaptable intervention in dose and frequency. In addition to assessing fidelity at the individual participant level, i.e., family members, applying the *receipt* and *enactment* dimensions enabled fidelity assessment at the unit level.

### Implementation fidelity: the REVERSE trial

REVERSE (“*pREVention and management tools for rEducing antibiotic Resistance in high prevalence Settings”*) is a stepped-wedge cluster-randomized hybrid effectiveness-implementation trial (type 2) aimed at assessing the effectiveness of infection prevention and control (IPC) and antibiotic stewardship (ABS) practice bundles on the health-acquired infections rate of 24 acute care hospitals in Europe^2^. REVERSE also involves assessing the effectiveness of tailoring as an implementation strategy, based on an additional randomization of participating sites to different implementation conditions. This required conceptualizing *tailoring fidelity* and operationalizing this concept for monitoring in the trial.

#### Tailoring fidelity

Tailoring has been characterized as a distinct implementation strategy [47] that involves *prospectively* identifying implementation barriers assumed to influence the implementation of an intervention. The goal is to inform the subsequent selection and design of implementation strategies assumed to address these pre-identified barriers and their planned and intentional application [48, 49]. While this implicit promise of *tailor-made* implementation appears appealing, the knowledge base for how tailoring can be systematically practiced, monitored, and evaluated remains limited. This gap has led to calls for more research in this area of implementation science [50, 51], including an in-depth exploration of *tailoring fidelity*, i.e., the actions or principles that constitute the core elements of tailoring which, when observed, confirm that such tailoring is taking place. This idea may appear counterintuitive. How can *firm* adherence be expected in the use of an implementation strategy that has context dependent *flexibility* as its core? Notwithstanding this tension, tailoring builds on generic principles – the prospective identification of determinants and the intentional determinant-informed selection and design of implementation strategies – making it possible to differentiate tailoring practice from less prudent attempts to select, develop, and apply implementation strategies. It is this conceptual challenge, the development of a tailoring fidelity framework, that is addressed in the REVERSE trial.

#### The REVERSE tailoring fidelity framework

**Fig. 3:**
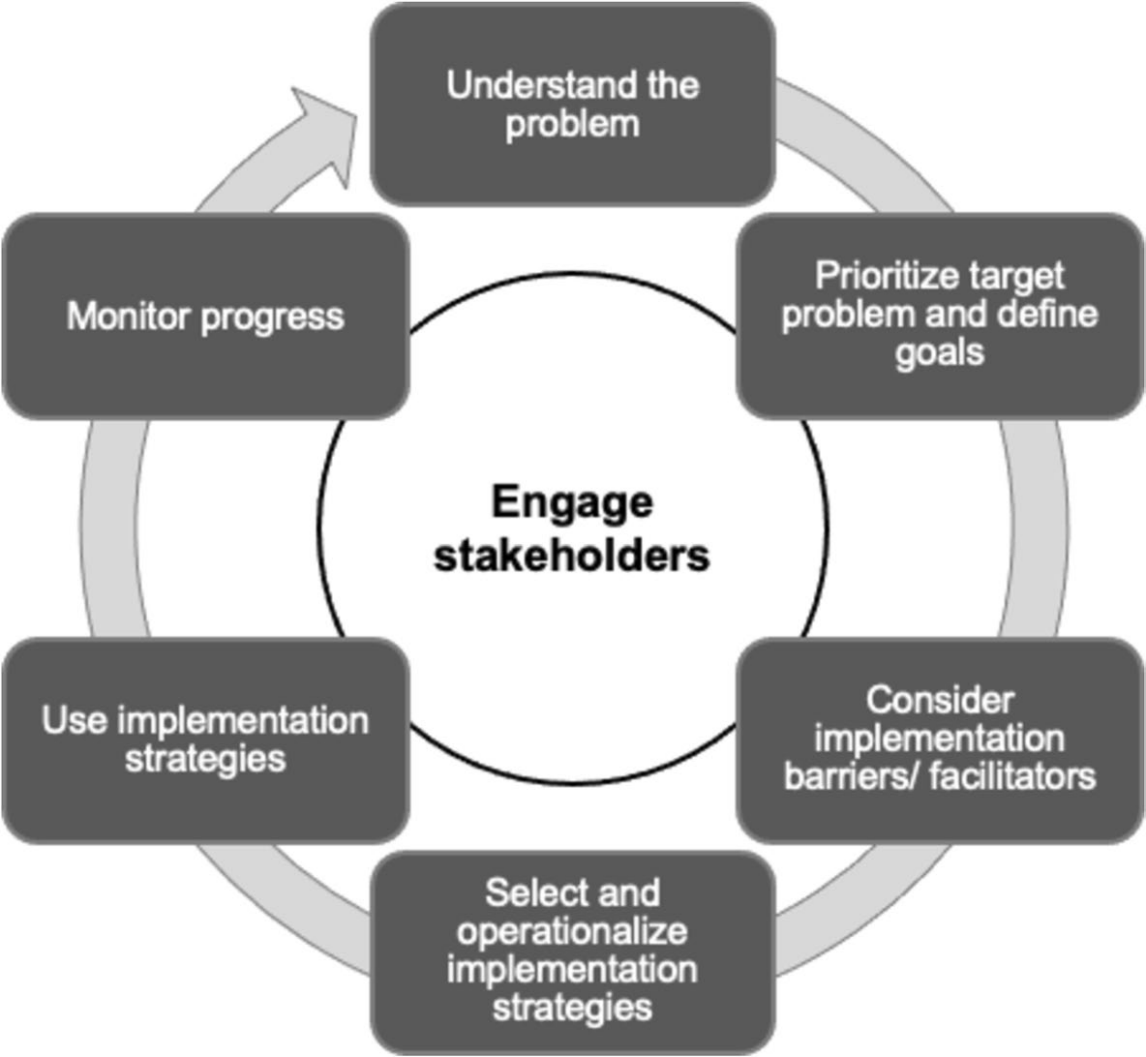
The REVERSE Improvement Cycle (RIC)

A pre-existing conceptual model of tailoring [52] was used to define tailoring key principles and to integrate these into what was labeled the REVERSE Improvement Cycle (RIC, Fig. 3): (1) prospective determinant identification; (2) intentional strategy-determinant matching; (3) context-sensitive operationalization of implementation strategies; and (4) implementation strategy use and impact assessment. These principles were combined with further steps involved in implementing REVERSE practice bundles, including prioritizing concrete IPC and ABS practices for implementation and considering approaches to stakeholder engagement throughout implementation.

The RIC represents a cyclical improvement logic that is widely used in quality improvement initiatives in healthcare [53–55], and which, as an implementation approach, was expected to resonate with study participants. It presents tailoring as an ongoing, potentially never-ending implementation strategy that requires paying continuous attention to changes in contextual factors relevant to implementation and to the needed adjustment of implementation strategies. This cyclical model forms the basis for teaching the principles of tailoring in workshops and online meetings with local implementation teams of participating hospitals working to implement the REVERSE practice bundles.

In a second step, the RIC was translated into the REVERSE implementation tool (RIT), a qualitative reporting instrument for participating sites to plan and document their implementation practice. Each step of the RIC is presented as one or multiple RIT questions for implementation teams to consider when preparing or documenting implementation actions. This includes thinking about the composition of the local implementation team, relevant stakeholders to engage, central determinants assumed to influence the implementation of a REVERSE practice, strategies for addressing these determinants, and indicators for monitoring own implementation practice. Sites are encouraged to use the RIT to plan their implementation activities, reflect on these activities, and document their implementation decision-making quarterly. Simultaneously, the RIT is a tool for the REVERSE implementation research team to follow sites’ tailoring efforts. It represents one element of the REVERSE tailoring fidelity assessment, which will be combined with RIT interviews with frontline hospital staff.

The assessment of RITs aims at understanding whether trial sites practice tailoring and is structured by the fidelity dimensions that are included in Demers et al.’s integrative conceptual fidelity framework [10]. Table 4 outlines how these dimensions were translated into questions, allowing the research team to assess local tailoring practice.

**Table 4:** The REVERSE tailoring fidelity framework

Tailoring Fidelity Dimensions - Implementers^*^		Tailoring Fidelity Dimensions - Receivers^∞^	
*Constructs and their definitions per Demers et al. (2021)*	*REVERSE targeting questions*	*Constructs and their definitions per Demers et al. (2021)*	*REVERSE targeting questions*
**Adaptation** The degree to which implementers deviate from planned implementation activities	Are any changes being made to the core tailoring model introduced to sites during initial workshops and subsequent support?	**Exposure** The actual dosage of implementation strategy received by receivers	To what degree do individual or groups of stakeholders acknowledge/recognize a prospective and dynamic approach to IPC/ABS implementation at their hospital?
**Adherence** The degree to which the implementation strategy is similar to what was planned	Is tailoring - in its entirety - used as planned, i.e., barriers are identified prospectively and matched with implementation strategies? Are only part of tailoring components used, e.g., strategies described but not linked to barriers?	**Enactment** The degree to which receivers understand and adhere to activities proposed by implementers	Do individual or groups of hospital stakeholders perceive that they have the ability, skill, and capacity to pursue a prospective and dynamic approach to IPC/ABS implementation in their own implementation practice?
**Differentiation** The degree to which the implementation strategy differs from implementation as usual	Is tailoring an implementation approach that is distinctly different from REVERSE hospitals’ usual IPC/ABS implementation practice?	**Reach** The number of receivers who received the implementation strategy	How many individual or groups of hospital stakeholders are exposed to the tailoring approach that the central hospital implementation team is using?
**Dosage** The amount of time spent on the implementation strategy (frequency, duration, length, intensity, or number of sessions)	How many “tailoring units”^+^ are delivered over which period of time? ^+^One “tailoring unit” = a newly detected determinant is prospectively matched with a distinct strategy.	**Responsiveness** The quality of the receivers’ response to the implementation strategy	To what degree do individual or groups of hospital stakeholders begin to pursue a prospective and dynamic approach to IPC/ABS implementation in their own implementation practice?
**Timeliness** The degree to which the implementation strategy is delivered at the right time	Is **initial** tailoring based on the prospective identification of implementation determinants? Is **ongoing** tailoring occurring when new determinants are detected, and/ or existing determinants change character or vanish?		
**Quality** The level of skill with which implementers apply the implementation strategy	What is the quality with which tailoring activities are described?		

^*^ **Implementers** = a hospitals’ REVERSE implementation team; ^∞^ **Receivers** = hospital stakeholders with whom the hospitals’ REVERSE implementation team collaborates in its efforts to implement REVERSE practice bundles

This table reflects that REVERSE tailoring fidelity will be explored at the level of tailoring *implementers* and tailoring *receivers*. Tailoring implementers are members of hospitals’ central IPC and ABS teams collaborating with frontline healthcare workers to facilitate the implementation of REVERSE practice bundles. These professionals function as internal implementation support practitioners [56, 57] and include IPC physicians, infectious diseases specialists, IPC nurses, microbiologists, pharmacists, and other healthcare workers specialized in preventing and controlling healthcare-acquired infections. They apply tailoring principles in their efforts to enable the local implementation of REVERSE practice bundles. Tailoring receivers are medical doctors, nurses, nurse assistants, cleaners, lab technicians, and other staff applying IPC or ABS quality standards in their daily work. They are the hospital stakeholders with whom IPC/ABS teams collaborate in their efforts to apply tailoring principles.

Hospitals’ IPC/ABS teams will share thirteen RITs throughout the REVERSE trial, leading to a maximum of 312 RITs documenting tailoring efforts. Each document will be assessed based on the questions in Table 4. Two REVERSE implementation research team members will review all RITs based on a list of indicators and attention points related to each targeting question. Of all RITS, 25% will be assessed in duplicate, and disagreements discussed in consensus meetings. The remaining RITs will be assessed by one team member only.

The RIT captures the implementation work of central IPC/ABS teams and, apart from the dimension *reach*, does not allow for a deeper examination of the degree to which tailoring receivers are exposed and respond to centrally initiated implementation efforts. In parallel with the administration of the RIT, healthcare frontline workers involved in REVERSE IPC/ABS implementation at each hospital will, therefore, be invited to interviews to explore to what degree exposure has taken place and has led to changed implementation practice at this level of service delivery. The targeting questions in Table 4 will be operationalized into guiding questions for semi-structured interviews to be held with a randomly selected sample of healthcare professionals. The findings from this assessment have the potential to help understand whether and how the use of tailoring in the REVERSE trial may influence trial results.

The experience from the REVERSE trial illustrates the complexity of conceptualizing fidelity to tailoring, a genuinely *adaptive* implementation strategy [21], and the considerable conceptual and empirical work required to assess this fidelity within the context of a hybrid type 2 trial with its inherent emphasis on implementation strategy fidelity.

### The intricacies of balancing firmness with flexibility

Together, the above case studies illustrate that researchers are confronted with multiple complexities when working to balance needs for both firmness and flexibility in conceptualizing, operationalizing, and evaluating intervention and implementation fidelity.

**First**, when researching (novel) complex interventions and implementation strategies, often little knowledge exists on the active ingredients that may trigger changes in implementation or health outcomes. Therefore, a certain degree of subjective decision-making may be involved in defining minimum fidelity requirements and creating clarity around where firmness is required and flexibility allowed. This also applies when evidence exists for the intervention and the implementation strategies under study, as there may still be a lack of knowledge on their combined application. One way to address this challenge is to develop a clear theory of change for an intervention and its implementation based on, e.g., implementation research logic models (IRLMs) [13, 58, 59]. IRLMs depict anticipated relationships between intervention components, contextual determinants, implementation strategies and their presumed mechanisms, implementation, and clinical outcomes. By necessitating thinking about *which* elements of an intervention or an implementation strategy are essential to achieve intended outcomes and about *how* these elements may create meaningful differences in individual, organizational, and system behavior and patients’ health and wellbeing, IRLMs create transparency around the conceptual and empirical foundations of defined fidelity requirements and their assessment. As empirical knowledge about the intervention and implementation strategies in focus increases, these requirements can then be revised and refined, and the room for firmness and flexibility defined with greater certainty.

**Second**, the operationalization and measurement of fidelity are always intervention- and implementation strategy-specific and study context-dependent. While efforts have been made to develop generic fidelity measures usable across multiple clinical interventions, even these remain target population-specific in that they address similar interventions for similar conditions pursuing similar goals [60, 61]. In most cases, intervention as well as implementation fidelity requirements and their assessment, therefore, depend on a full conceptual development that is adjusted to the intervention and delivery setting in focus of a study. Hence, non-negotiable core intervention or implementation strategy elements viewed as requiring firmness when used with a particular target population in one setting may be adjustable and allow for flexibility when applied with another target population in a different setting. This possibility makes it critical for researchers to re-think fidelity concepts with every new study.

Furthermore, and of particular importance to implementation scientists, the design of such a study may represent a further contextual factor to consider when measuring fidelity. Depending on the type of hybrid study chosen [39], its primary focus will be either the intervention, the implementation strategy, or both, leading to different emphasis on measuring intervention and implementation fidelity. In a hybrid type 1 study, with the primary aim of determining intervention effectiveness, measuring intervention fidelity is essential, whereas implementation is primarily assessed from an exploratory perspective. There may, therefore, be limited room for flexibility and a focus on firmness when assessing intervention fidelity [44, 62]. In a hybrid type 2 or 3 trial, the assessment of implementation strategies is in focus. Consequently, the room for continuously adjusting these strategies may be limited to ensure a thorough evaluation of their effect.

To address this challenge of fidelity operationalizations being intervention- and implementation strategy-specific, some scholars have suggested moving away from intervention protocols that emphasize the often highly protocolled and detailed *forms* in which interventions should be delivered to instead focus on *function* descriptions as the key referent for firm fidelity requirements [63]. For an implementation strategy such as *tailoring*, prospective determinant identification and purposeful determinant-strategy matching represent such essential functions ensuring that implementation work considers and addresses the contextual conditions of a local setting. These functions could be pursued in various forms, e.g., through regular staff meetings, surveys, expert interviews, analysis of written materials, and similar approaches that implementers could select based on what is appropriate and feasible in their setting. In this way, the form-function model can enhance the flexibility with which interventions and implementation strategies are designed to fit a local, ever-changing context. Integrating this form-function thinking into the use of fidelity frameworks to develop fidelity measures can help make these measures more robust through nuanced thinking about, e.g., what appropriate *timeliness*,* quality*, or *exposure* for delivering an intervention or implementation strategy would mean.

**Third**, fidelity concepts continue to focus on individual or group behavior and actions. However, individuals and groups always operate in broader contexts that influence their implementation, an influence acknowledged in, e.g., the recently updated Carroll fidelity framework, now also including *organizational and cultural context* [64]. Here, context is viewed as a fidelity moderator, affecting the strength of adherence achieved in any given setting. If contexts are not supportive of fidelity efforts, individuals and groups striving to comply with intervention or implementation fidelity criteria may still be unsuccessful. While viewing contextual factors as moderators in this way clarifies implementation processes, it also represents a limitation in that context is treated as *a given*. Instead, it could be valuable to consider how organizational and system-level requirements could be better integrated into conceptualizations of fidelity. When viewing individuals as being embedded in teams, organizations, and systems, their ability to stay firm and be flexible when needed may depend on, e.g., leadership support, dedicated work hours, or formally recognized staff roles – factors that could be defined as additional organizational or system fidelity requirements, to indicate clearly that fidelity never occurs in a vacuum. In a recent publication on IPC implementation, this relationship was described as a cascading logic model [57] unfolding across three levels, combining intervention fidelity at the frontline healthcare worker level with implementation fidelity considerations at the level of direct superiors and that of hospital management, highlighting that the organization surrounding frontline implementers has levers to provide fidelity supports. To the degree such supports can be defined as vital functions for building fidelity, these could be integrated into multi-level fidelity concepts that go beyond the individual and assign the organization a degree of fidelity accountability. Implementation strategies developed to be applied at the organizational or community level to, for example, enhance implementation leadership, climate, or culture [65–67] allow for testing this relationship.

**Finally**, with contemporary conceptualizations of fidelity centered on function rather than form, thereby moving away from simple checklist-based assessments of firmness and instead creating room for intervention adaptation and implementation strategy tailoring, activities representing intervention or implementation fidelity often contain a substantial *process* component. In balancing firmness with flexibility, implementers increasingly require capacity building, process facilitation, or problem-solving skills. Fidelity is, therefore, not easily built and not easily assessed continually and objectively [68–70]. Its assessment will require regular training and exchange between researchers, implementation support practitioners, and frontline implementers, thereby depending on the use of considerable resources, as illustrated through the case examples shared above. While these resources may be available as part of a research trial, there is typically much less room for an ongoing fidelity assessment in routine healthcare settings unless pragmatic approaches can be found. This raises the question of how low one can go in finding this pragmatic level at which fidelity achievement is possible, data collection and feedback loops doable, and meaningful data can be generated, e.g., to inform decision-making on encouraging greater firmness or flexibility among implementers.

Intensifying contemporary fidelity research focusing on intervention *and* implementation fidelity is pivotal. Theoretically, there remains a need to understand better how an appropriate balance between firmness and flexibility in intervention delivery and implementation strategy use may add to improved implementation, service, and patient outcomes and how this balance can be obtained using the least possible resources and taking into account influences at the individual, organizational and system level. Empirically, there continues to be a need for complex fidelity studies examining the contribution of intervention and implementation fidelity, separately and combined, to different outcomes. Furthermore, greater knowledge is needed about practices for building, evaluating, and maintaining intervention and implementation fidelity. This includes work to explore if and how generic and pragmatic fidelity measurement instruments can be developed for the routine assessment of intervention and implementation fidelity in various healthcare research and service settings.

## Conclusion

In accepting the ever-changing complexity of routine healthcare settings, the common understanding of fidelity in the field of (complex) health intervention research and implementation science has become more nuanced in recent years. A formerly dominant emphasis on firm compliance to form-focused elements of interventions has been replaced by a stronger recognition of the need for balancing firmness with flexibility in intervention implementation. Simultaneously, the domain of implementation strategies has emerged as a new frontier in the fidelity debate, raising questions about if and how the concept of fidelity may be applied and linked to that of intervention fidelity.

For researchers, intervention developers, and practitioners utilizing fidelity to monitor or evaluate intervention delivery and implementation, these developments make it pertinent to think thoroughly about conceptualizing intervention and implementation fidelity in their specific context. This includes defining the core components of clinical interventions and the strategies used for their integration into practice, thereby clearly articulating the boundaries between intervention and implementation. It also implies specifying the assumed mechanisms of desired outcomes, explaining the degrees of firmness and flexibility that characterize a given fidelity model. In this way, contemporary fidelity work is a *balancing act* emphasizing the need for healthcare researchers and professionals to continually consider how contextual conditions change and require adjustments in ongoing intervention implementation. Being agile as a researcher, implementer, or organizational leader requires capacity, skill, and supportive tools, e.g., in the form of fidelity frameworks that reflect the complex realities of fidelity not only being an individual but just as much an organizational and system responsibility, or in the form of a stronger knowledge base on how to develop and apply intervention and implementation fidelity measures that meet the needs of busy healthcare professionals and organizations. Continuing to build this fidelity knowledge base and enhance our understanding of how to attune to firmness and flexibility requirements is a key task for implementation scientists.

## Data Availability

Not applicable.

## Footnotes

1
https://www.ifis.uzh.ch/de/Lehre-Forschung/Forschung/Projekt-FICUS-Trial.html.

2
https://www.reverseproject.eu/.

## Abbreviations

ABS: Anti-Biotic Stewardship
FICUS: Family support intervention in Intensive Care UnitS
FSI: Family Support intervention
ICU: Intensive care unit
IPC: Infection Prevention and Control
REVERSE: pREVention and management tools for rEducing antibiotic Resistance in high prevalence SEttings
