# Correction: Firm, yet flexible: a fidelity debate paper with two case examples

**DOI:** 10.1186/s13012-024-01411-6

**Published:** 2025-01-06

**Authors:** Bianca Albers, Lotte Verweij, Kathrin Blum, Saskia Oesch, Marie-Therese Schultes, Lauren Clack, Rahel Naef

**Affiliations:** 1 Institute for Implementation Science in Health Care, Universitätstrasse 84, Zurich, 8006 Switzerland; 2 Center of Clinical Nursing Science, University Hospital Zurich, Zurich, Switzerland; 3 Division of Infectious Diseases and Hospital Epidemiology, University of Zurich and University Hospital Zurich, Zurich, Switzerland

**Correction** **:** **Implementation Sci 19, 79 (2024)**

**https://doi.org/10.1186/s13012-024-01406-3**

In the Original Article [1], a note indicating the equal contribution of authors Bianca Albers - Lotte Verweij and Lauren Clack - Rahel Naef was erroneously removed during typesetting.

The note should be as follows:

**Bianca Albers and Lotte Verweij shared first authorship**

**Lauren Clack and Rahel Naef shared last authorship**

The original article has been corrected.
